# Rectal retractor in prostate radiotherapy: pros and cons

**DOI:** 10.1186/s13014-022-02176-2

**Published:** 2022-12-09

**Authors:** Hamed Ghaffari, Arezoo Mehrabian

**Affiliations:** 1 Department of Medical Physics, School of Medicine, Iran University of Medical Sciences, Tehran, Iran; 2 Department of Radiation Oncology, Imam Hassan Mojtaba Hospital, Dezful University of Medical Sciences, Dezful, Iran

## Abstract

Dose escalation in prostate radiotherapy (RT) have led to improved biochemical controls and reduced the risk of distant metastases. Over the past three decades, despite technological advancements in RT planning and delivery, the rectum is a dose-limiting structure in prostate RT owing to the close anatomical proximity of the anterior rectal wall (ARW) to the prostate gland. RT-induced rectal toxicities remain a clinical challenge, limiting the prescribed dose during prostate RT. To address the spatial proximity challenge by physically increasing the distance between the posterior aspect of the prostate and the ARW, several physical devices such as endorectal balloons (ERBs), rectal hydrogel spacers, and rectal retractor (RR) have been developed. Previously, various aspects of ERBs and rectal hydrogel spacers have extensively been discussed. Over recent years, given the interest in the application of RR in prostate external beam radiotherapy (EBRT), this editorial will discuss opportunities and challenges of using RR during prostate EBRT and provide information regarding which aspects of this device need attention.

As demonstrated in randomized clinical trials, dose escalated/hypofractionation regimens in prostate radiotherapy (RT) have led to improved biochemical controls and reduced the risk of distant metastases [1–4]. Over the past three decades, despite technological advancements in RT planning and delivery such as intensity-modulated radiotherapy (IMRT) and image-guided radiotherapy (IGRT), the rectum is a dose-limiting structure in prostate RT owing to the close anatomical proximity of the anterior rectal wall (ARW) to the prostate gland [5, 6]. RT-induced rectal toxicities remain a clinical challenge, limiting the prescribed dose during prostate RT. It has been demonstrated that there are dose-response relationships for long-term rectal toxicity following prostate RT. To address the spatial proximity challenge by physically increasing the distance between the posterior aspect of the prostate and the ARW, several rectal sparing devices such as endorectal balloons (ERBs), rectal hydrogel spacers, and rectal retractor (RR) have been developed [7–11]. Previously, various aspects of ERBs and rectal hydrogel spacers have extensively been discussed [7, 9, 10, 12]. Herein, given the interest in the application of RR in prostate external beam RT (EBRT), this editorial will discuss opportunities and challenges of using RR during prostate EBRT.

The purpose of applying RR is mainly to improve the daily reproducibility of the rectal wall (RW) position, reduce prostatic motion, and improve rectal dosimetry by pushing the posterior and lateral RW away from the high-dose region. This device decreases RW doses by physically separating the RW from the prostate, thereby resulting in a reduction in rectal toxicity rates. Several studies have already shown that using RR can significantly reduce the absolute and relative dose-volume parameters of the rectum during prostate three-dimensional conformal RT (3DCRT), IMRT, proton therapy, and stereotactic body RT (SBRT), both as primary treatment and post-prostatectomy salvage RT [13–17]. In a comparative study, it has been found that both RR and hydrogel spacer reduce rectum V30-80% during prostate SBRT; however, RR was able to further reduce the rectal volume receiving low and intermediated doses compared to hydrogel spacer [16]. It is also important to point out that information regarding anal wall sparing effect of RR has not yet been published.

An interesting benefit of this device is that retraction of the rectum can significantly reduce the ARW doses [13, 14]. When the RR retracts the rectum, the ARW is slightly displaced from the prostate, and also the rectal retraction changes rectal shape, consequently reducing the volume of the ARW receiving high doses of radiation. It has been seen that using a RR can increase the space between the clinical target volume (CTV) and the ARW on sagittal CT images [14]. Of note, assessment of the space between the posterior borders of the CTV and the ARW on sagittal CT images only may be biased by the low resolution of the CT scan compared to MRI; however, separating ARW from the prostate at different levels (i.e. sagittal, coronal, and axial) on MRI would be of interest.

It is important to highlight the fact that the most important argument to apply RR is to eventually alleviate radiation-induced rectal toxicities. In a single-arm study, using RR during dose-escalated prostate IGRT resulted in improving acute rectal toxicity rates, and no acute grade ≥ 2 rectal toxicities during treatment were found [13]. In a comparative clinical study, Arefpour et al. compared 18 patients with and 18 patients without RR, treated with five-field 3DCRT to a total dose of 70 Gy in 35 fractions [18]. The rate of acute toxicity was similar between groups during treatment or at 3 months after RT. At 12 months, patients treated with RR experienced significantly less late grade ≥ 1 rectal toxicity, with no grade ≥ 2 toxicity. Late grade 2 rectal toxicity was experienced by 3 patients in the control group [18]. More recently, a study from Tampere University Hospital, Tampere, Finland has reported that the use of RR does not reduce acute rectal toxicities in patients treated with conventionally fractionated RT (78/2 Gy) and moderate hypofractionated RT (60/3 Gy) [19]. Also, it has been found that patients treated with RR had more frequent rectal haemorrhages, which resulted in worse quality of life (QOL) at the end of RT [19]. Any RR acute rectal toxicity benefit may have been masked by repeated physical placement of RR daily irradiation. It is noteworthy that anal and rectal mucous become sensitive after daily irradiation, and daily retraction of the rectum intensifies the aforementioned issue and may result in rectal irritation and rectal haemorrhages. Besides, unintended small bowel or sigmoid colon irradiation with low doses may mask the impact of RR on reducing acute rectal toxicity. To date, there are no randomized trials reporting long-term rectal toxicities with RR in-place; therefore, randomized clinical studies with long-term follow up are warranted to evaluate whether the rectal dose-sparing impact of RR translates into reduced late rectal toxicities and improved QOL.

As stated earlier, applying RR pushes RW (both ARW and posterior rectal wall (PRW)) away from the CTV; therefore, it alters RW dose distribution and leads to a shift in ratio between low- and high-dose irradiated RW surface areas that may have a positive impact on mucosal regeneration [20, 21]. Moreover, retraction and stretching of the RW may have an effect on vasculature; a reduced blood volume in the rectum can cause hypoxia, which increases radioresistance of the rectum.

It is worthwhile to mention that the volume of the rectum is increased using a RR; however, the volume of the RW is constant before and after the insertion of the RR [22]. Therefore, RW delineation is essential to clear the impact of a RR on the rectal dose-volume parameters during prostate RT.

An additional potential benefit of RR is prostate-stabilizing effect. Previous studies have demonstrated the RR has a strong potential to decrease intra-fraction prostate displacement and increase inter-fraction RW position reproducibility [15, 23–25]. Comparative studies using various IGRT systems such as pre- and post-treatment cone-beam CT, Cine-MRI, and Kilovoltage intra-fractional monitoring have indicated that application of the RR reduces intra-fractional prostate motion compared to patients treated without RR [23–25]. However, the prostate immobilization effect of the RR has not confirmed by a comparative study using a single measurement point provided by RayPilot® electromagnetic real-time tracking system [26]. The most important reason for this discrepancy can be associated with various bowel preparation regimens prior to daily treatment. It is worthwhile to mention that previous studies are limited due to the restriction on motion management. The aim of the motion management, i.e. decreased radiation induced-rectal toxicity and increased efficacy by a lower target miss rate were not covered. Thus, prospective studies are expected to clarify whether the prostate immobilizing impact of RR can result in using smaller planning target volume (PTV) margins and thereby further sparing of the rectum.

There are several concerns regarding daily insertion of RR including, patient tolerability and convenience, procedure-related toxicity, and workload. At the first glance, daily application of RR can cause anal and rectal irritation and as a result, this device cannot be used throughout all treatment sessions with a conventionally fractionated RT regimen. Hence, previous clinical studies applied daily insertion of RR during the cone-down 15–20 and 10 treatment fractions in conventionally fractionated and moderate hypofractionated RT, respectively [13, 18, 19]. In a study, patients treated with conventionally fractionated IMRT (80/2 Gy) and a RR in-place experienced a mild pain according to the visual analog score [14]. The mean pain score with RR was 2.7 [14]. As reported in studies, patients experienced some discomfort associated with daily insertion of RR owing to the rectal retraction, but it was well tolerable [13, 14, 18]. In the PROMETHEUS study, it has been reported that discomfort with RR was moderate in 35% and severe in 14% of men [27]. No severe complications, i.e. severe anal irritation or rectal bleeding, have been reported with daily RR use [13, 14]. Only one study reported that 2 out of 35 patients could not tolerate the insertion of RR [16]. Several factors can potentially increase patient tolerability including, patient willingness and collaboration, staff motivation, and gradually retraction, as well as using lubricant gel or lidocaine jelly. Using RR is limited in patients with pre-existing anorectal disorders (*e.g.* hemorrhoids, anal fissure, and fistula) [13, 14, 18]. Daily insertion of RR into the rectum is time-consuming and requires an additional time of approximately 3–4 minutes per treatment session [13]. If RR placement is intolerable for the patient during any of the treatment fractions throughout the entire RT course, this results in a need to re-plan the radiation treatment and/or delay RT delivery, further increasing workload. It is noteworthy that the rectal rod sterilized and capped with disposable condom is first gently placed in the patient's rectum by a physician at CT-planning to ensure a proper position of RR and patient’s tolerance. In RT sessions, the insertion of RR is performed by a physician-directed healthcare professional. To increase the efficacy of the RR in retracting the rectum and the reproducibility of the RW position, patients must instruct to have a proper rectal preparation before the planning CT-scan and RT sessions.

Another potential advantage of RR is in vivo RW dosimetry with various types of dosimeters such as MOSkin detectors, GafChromic EBT3 film, and PTW-31014 Pinpoint chamber for patient-specific quality assurance [13, 25, 28]. Economically, the RR is one-off department purchase and is used for all patients; therefore it does not impose a high cost on the health care system. Additionally, the RR may potentially enable the development of RT treatment protocols investigating hypofractionation or ultra-hypofactionation regimens or hypofractionated/dose escalated RT with simultaneously integrated boost. Studies on RR in post-prostatectomy RT setting are very rare. To date, however, only one case report has studied the effect of RR on rectal dosimetry during post-operative RT, reporting promising results [17]. Further clinical studies will be required to clarify the role of RR in post-prostatectomy RT. RR as a rectal sparing device can also be incorporated with prostate re-irradiation. Clinical studies are awaited to evaluate the feasibility of the application of RR in prostate re-irradiation.

It is interesting to address pros and cons other rectal sparing devices, i.e., ERBs and rectal hydrogel spacers, to compare various aspects associated with each technology. An old rectal sparing device is ERB insertion, a minimally invasive method for decreasing anorectal radiation doses during prostate RT. The deflated ERB is inserted into the rectum and then inflated with air or water, resulting in increasing the distance of PRW from the high-dose regions. Although utilizing an ERB can reduce the PRW doses, it increases the ARW volume receiving high doses of radiation because pushes the ARW toward the prostate. The most important benefit of ERB technique is that it significantly restricts intra-fraction prostate motion [8]. However, day-to-day reproducibility of ERB position is challenging, and variations in ERB placement depth can cause prostate deformation that consequently affects dosimetric outcomes [8, 11]. Moreover, repeated insertion of ERB may cause adverse rectal tissue reaction and anorectal irritation on patients owing to direct contact with the rectal mucosa. Hence, the insertion of ERB into the rectum is facilitated using lidocaine ointment and lubricant jelly. The placement of ERB is well tolerated without severe complications; however, some patients do not tolerate ERB insertion throughout the entire course of RT [11]. The insertion of ERB is contraindicated in patients with pre-existing anorectal diseases. Daily insertion of ERB into the rectum is time-consuming and adds approximately 3 min additional time to routine treatment setup [11]. The use of ERB can also provide the opportunity for in vivo dosimetry for rectal dose verification during prostate RT [29]. In post-prostatectomy RT setting, using ERB cannot significantly reduce anorectal doses because the inflated ERB pushes ARW toward the prostate bed owing to missing counterfort of the prostate gland in this scenario [11]. It is important to mention that daily insertion of ERB is performed under uniform guidelines. A daily disposable ERB should be applied for hygiene reasons. From an economic point of view, one ERB can be used per patient throughout the entire course of prostate RT with the use of a disposable condom in each fraction.

More recently, an innovative strategy, SpaceOAR hydrogel, has been introduced in clinical practice with the intent to decrease RT-induced rectal toxicity during prostate RT [7, 8, 11]. The hydrogel is injected between the Denonvilliers’ fascia and the rectum about one week prior to RT to separate the ARW from the prostate and consequently reduce the radiation dose delivered to the ARW. Using SpaceOAR hydrogel significantly increases the prostate-rectum separation, a mean separation of approximately 10 mm, which results in significantly reduced rectal volumes irradiated to high doses [7]. Disadvantage of this method is associated with the invasive implantation procedures that can cause some complications. Hydrogel is injected under general or local anaesthetic via a transperineal approach with transrectal ultrasound guidance [7, 11]. The injection of SpaceOAR hydrogel is time-limited because it polymerized at few second; therefore in situ positioning correction of the hydrogel is not usually possible [11]. The injection of hydrogel spacer is safe. However, rarely, some complications following SpaceOAR hydrogel implantation have been observed including, severe anaphylaxis, acute pulmonary embolism, prostatic or perineal abscess and sepsis, rectal wall erosion, rectal ulceration and fistula, and recto-urethral fistula [7, 11]. In contrast to RR and ERB, there is a growing body of evidence that supports the application of SpaceOAR hydrogel during prostate brachytherapy with promising results [7]. The injection of SpaceOAR hydrogel does not serve as a stabilizing device for prostate motion [8]. Regarding post-prostatectomy RT, the benefit of the implantation of hydrogel spacer is controversial [30]. However, Pinkawa et al. proposed that this approach can be used for specifically selected cases [31]. There are concerns regarding the cost-effectiveness of the routine use of SpaceOAR hydrogel [11, 32]. The cost of SpaceOAR hydrogel is approximately 1500 € (expressed in euros (€)) [32]. It has been reported that the routine SpaceOAR use is not cost-effective [32]. Further studies are awaited to confirm the cost-effectiveness of this approach to elucidate which subset of prostate patients would benefit from the implantation of hydrogel to decrease rectal toxicities in a cost-effective manner.

A growing body of literature supports that the use of SpaceOAR hydrogel meaningfully and significantly decrease both the rectal doses and the long-term RT-induced rectal toxicities during prostate IMRT, SBRT and proton therapy [33–35]. Most existing literatures on RR focus on the dosimetric effects, but data on patient- and physician-reported toxicities with the use of RR are scarce. Regarding the role of RR in prostate proton therapy and hypofractionated protocols, more data are awaited to confirm the efficacy of this approach to reduce long-term rectal toxicities. The ERB was introduced early in the 3D era without image-guidance and the dosimetric advantages of this method have clearly been indicated by 3DCRT; however, improved rectal dosimetry with ERB was not always reproduced with the advent of intensity-modulated techniques with imaging guidance [36]. Also, using ERB significantly increased the volume of the RW receiving high radiation doses during prostate SBRT [37]. Regarding ERBs, there is no level 1 evidence to support their utility in decreasing RT-induced rectal toxicity or high doses to the ARW [38].

Interestingly, many patients with prostate cancer are cured with both surgery and RT and, even without rectal sparing devices; a very low rate of adverse events requiring intervention has been reported. Therefore, any recommendation to implement these devices in routine clinical practice requires careful consideration of the need and evidence of toxicity and clinical benefits of rectal sparing devices.

In summary, using RR may represent a new horizon for prostate EBRT delivery. This device along with state of the art RT delivery techniques can be a game changer in prostate cancer. Although clinical experience with this device is scant but prostate RT treatments with RR in-place seem feasible and tolerable to patients. Therefore, collection of clinical evidence in well-designed clinical trials is warranted to fully explore the true potential clinical benefits and cost-effectiveness of this innovative technology. It is also interesting to evaluate whether various rectal sparing devices have the same clinical benefits in the era of modern EBRT for prostate cancer.

## Data Availability

No data generated.

## Abbreviations

RT: Radiotherapy
IMRT: Intensity-modulated radiotherapy
IGRT: Image-guided radiotherapy
ARW: Anterior rectal wall
ERB: Endorectal balloon
RR: Rectal retractor
EBRT: External beam radiotherapy
RW: Rectal wall
3DCRT: Three-dimensional conformal radiotherapy
SBRT: Stereotactic body radiotherapy
CTV: Clinical target volume
QOL: Quality of life
PRW: Posterior rectal wall
PTV: Planning target volume
